# Plutonium mobility and reactivity in a heterogeneous clay rock barrier accented by synchrotron-based microscopic chemical imaging

**DOI:** 10.1038/s41598-024-53189-8

**Published:** 2024-02-07

**Authors:** U. Kaplan, S. Amayri, J. Drebert, D. Grolimund, T. Reich

**Affiliations:** 1 Department of Chemistry, Johannes Gutenberg-Universität Mainz, 55099 Mainz, Germany; 2 Swiss Light Source, Paul-Scherrer-Institut, 5232 Villigen PSI, Switzerland

**Keywords:** Biogeochemistry, Pollution remediation

## Abstract

The long-term safe disposal of radioactive waste corresponds to a challenging responsibility of present societies. Within deep geological waste disposal concepts, host rocks correspond to the ultimate safety barrier towards the environment. To assess the performance of such barriers over extended time scales, mechanistic information on the interaction between the radiotoxic, long-lived radionuclides like plutonium and the host rock is essential. Chemical imaging based on synchrotron microspectroscopic techniques was used to visualize undisturbed reactive transport patterns of Pu within pristine Opalinus Clay rock material. Pu^+V^ is shown to be progressively reduced along its diffusion path to Pu^+IV^ and Pu^+III^ due to interaction with redox-active clay rock constituents. Experimental results and modeling emphasize the dominant role of electron-transfer reactions determining the mobility of Pu in reactive barrier systems. The effective migration velocity of Pu is controlled by the kinetic rates of the reduction to Pu^+IV^ and Pu^+III^ and the redox capacity of the involved electron donor pools. To advance our predictive capabilities further, an improved understanding of the nature and capacity of redox-active components of the reactive barrier material is fundamental. The findings represent an essential contribution to the evaluation of the long-term safety of potential nuclear waste repositories and have implications regarding the development of effective geological disposal strategies.

## Introduction

The long-term safe disposal of radioactive waste corresponds to a challenging responsibility of present societies. Concepts based on a multi-barrier repository in deep geological formations represent a promising class of disposal strategies^1–4^. Within these concepts, the host rock corresponds to the ultimate safety barrier towards the environment. To assess the performance of such final barriers, detailed information on the interaction between the different radiotoxic, long-lived radionuclides and the host rock is essential. Furthermore, to allow for reliable long-term predictions concerning the spreading of long-lived radionuclides, it is fundamental to identify and understand the physico-chemical reactive (solute) transport mechanisms along the entire transport paths through all the different barriers.

Based on inventories and their long half-lives, plutonium (Pu) and other actinides are the major contributors to the radiotoxicity of spent nuclear fuel in a deep geological repository after a storage period of several hundred years^5,6^. After its discovery in the 1940s, Pu was found to be a distinctive chemical element. Twenty Pu isotopes have been identified. Concerning wastes arising from nuclear power generation, the isotopes ^239^Pu and ^242^Pu with half-lives of 2.41 × 10^4^ years and 3.75 × 10^5^ years, respectively, are the most important ones due to their high radiological toxicities and long-term persistence in the environment. Considering its chemical properties, Pu is a highly redox-sensitive element with a resulting complex chemical behavior. Under conditions relevant to waste repositories as well as to near-surface environments, Pu can exist in aqueous solutions in several oxidation states simultaneously^7,8^. The electronic states and chemical speciation are strongly dependent on the local chemical conditions such as pH, Eh, complexing ligands, and ionic strength. This ability to exist in multiple oxidation states stems from similarities in reduction potentials and disproportionation^9–11^.

Based on its complex geochemistry, the mobility of Pu in natural subsurface systems is potentially controlled by a broad range of geochemical reactions, including sorption reactions, precipitation, redox reactions, complexation, as well as the formation of poly-nuclear and colloidal species^12^. However, the corresponding reaction rates and equilibrium states differ significantly between the different oxidation states^13^. Accordingly, the oxidation state of Pu has to be considered as the primary factor in determining its mobility in the subsurface environment. In general, Pu^+III^ and Pu^+IV^ show low solubility and a strong chemical affinity for mineral surfaces (strong adsorption or surface precipitations) resulting in a considerable retardation of the Pu spreading, whereas Pu^+V^ and Pu^+VI^ exhibit a low tendency for hydrolysis, precipitation, and sorption on natural minerals. As a consequence, the oxidized species +V and +VI exhibit a pronounced mobility in subsurface systems. Overall, the migration behavior of Pu in the environment depends strongly on its oxidation state which is significantly affected by the geochemical characteristics of the host rock material, e.g., its mineralogy and coupled pore water chemistry^14–16^.

Based on their very low hydraulic conductivity and their high retention capabilities, argillaceous rocks such as Opalinus Clay (OPA) are currently examined by several European countries as potential host rocks for high-level radioactive waste repositories^4,17,18^. From a geochemical perspective, argillaceous rocks represent complex and heterogeneous porous media. The mobility of reactive solutes in such media is the result of a complex interplay of multiple reactions involving a broad variety of mobile and immobile reactive species. Furthermore, the level of complexity is increased considerably for redox-sensitive nuclides such as Pu. Several studies investigated the geochemical reactivity between Pu and individual, pure clay mineral phases considered as potentially reactive components of clay rocks (e.g., kaolinite, illite, montmorillonite, different iron oxides, calcite or chukanovite)^19–27^. All results showed that the affinity for mineral surfaces depends on the oxidation state of Pu, solution pH, ionic strength, and the surface characteristics of the sorbent.

As already pointed out, the mobility of Pu in a geochemically complex and heterogeneous material such as OPA is the result of a subtle network of coupled, hierarchical processes. The different redox species are linked to each other through electron transfer reactions. Depending on the electronic state, pronounced differences in terms of reactivity are expressed. Complexation with various complexing agents in solution, surface complexation with different components of the porous medium, as well as formation of polynuclear species or solid phases (precipitation), all these reactive processes depend strongly on the redox state^28^. Already subtle changes in the chemical environment (e.g., pH, Eh, or pore water composition) will trigger a cascade of successive reactions.

Nonetheless, only a limited number of studies addressed the environmental reactivity between Pu and pristine natural barrier materials like bentonite and argillaceous rocks, representing geochemically complex multicomponent systems. Amayri et al.^29^ studied the sorption of Pu and other actinides on OPA material by batch sorption experiments. This study showed that the affinity for the solid surfaces depends indeed strongly on the oxidation state of the actinides. The order of sorption strength at pH 7.6 is shown to be (+IV ≈ +III) >  > (+VI ≈ +V); accordingly radionuclides in the tri- and tetravalent states sorb strongest and the ones in the penta- and hexavalent states sorb weakest. Begg et al.^30^ investigated the sorption behavior of Pu^+IV^ and Pu^+V^ to FEBEX bentonite over impressively large concentration ranges (10^–7^–10^−16^ M for Pu^+IV^). For both Pu species a linear sorption behavior was observed. In the case of Pu^+V^, a time-dependence of the adsorption strength pointed towards reduction of Pu^+V^ on clay minerals during the batch studies. Kaplan et al.^31^ studied the sorption of Pu during interaction with intact OPA material. Based on multimodal microscopic chemical imaging, this study documented a pronounced impact of geochemical heterogeneities regarding the local reactivity of the natural barrier material. Spatially resolved X-ray absorption spectroscopy documented a reduction of the mobile Pu^+V^ and Pu^+VI^ to the less mobile Pu^+IV^ within the argillaceous rock material. Based on simultaneous micro-diffraction imaging, kaolinite was identified as a reactive mineral phase with considerably enhanced affinity for Pu^+IV^. Their findings provide strong evidence that reduction and immobilization does not occur as linked processes on a single reactive phase but as decoupled, subsequent, and spatially separated reactions involving different phases of the OPA^31^.

Reactive transport experiments involving Pu as reactive tracer and real barrier materials are very challenging and only sparse experiments are published so far. Corresponding studies report mostly one-dimensional diffusion profiles with rather coarse spatial resolution. Rämebeck et al.^32^ and Albinson et al.^33^ investigated the migration of Pu into compacted bentonite. Both studies used fuel material as Pu source. Ashida et al.^34^ studied the leaching and subsequent diffusion of Pu out of doped glass into compacted bentonite. For all three studies the Pu release by dissolution from the solid sources corresponded to a rate limiting step biasing the observed diffusion profiles as the condition and flux at the source (‘inlet’) boundary are not well defined or known. Further, the redox state of the diffusing Pu species was not controlled or determined in these studies. To the best of our knowledge, two-dimensional transport patterns of Pu spreading in an argillaceous host rock or alternative reactive barrier material have not yet been reported.

As a review of the literature reveals, the geochemical reactivity of barrier materials is commonly studied by disruptive batch studies yielding as observable the superimposed outcome of all occurring geochemical processes. Similar, considering solute transport studies like in-diffusion experiments, the geochemical processes occurring within the porous medium are commonly derived indirectly based on the observation of flux outside or at the boundary of the rock material. Consequently, both approaches yield highly valuable, but empirical, nonmechanistic knowledge concerning the geochemical reactivity of the porous media. However, without a better mechanistic understanding at the molecular level, the advance of our ability to model transport behavior and to achieve high confidence in predicting long-term transport is considerably limited.

Yet, the analysis of undisturbed reactive transport pattern expressed within the porous medium can contribute to enhance our mechanistic understanding. By complementing disruptive batch studies by in-situ microspectroscopic investigations of reactive transport pattern, two main benefits arise. First, microscopic, multimodal chemical images of reactive transport patterns allow to deconvolute the chemical complexity. Obtaining spatially resolved chemical information and the corresponding spatial context opens up the possibility to correlate the local chemical nature and state of the porous medium with the expressed reactive transport pattern. As a potential result, new phenomena, relevant mechanism, and reactive components can be identified (e.g., Kaplan et al.^31^). Second, reactive transport patterns correspond to invaluable data to validate reactive (solute) transport models. The observed pattern is the result of multiple coupled reactions evolving over a defined time period. To predict the spatial distribution of the reactive tracer, models must include the predominant reaction mechanisms and corresponding reaction rates.

The present study visualizes the in-situ micro-scale reactive transport of Pu within pristine Opalinus Clay rock material under ambient air conditions. For almost one month, aqueous plutonium Pu^+V^ was entering from a reservoir the pore space of the rock, migrating further into the rock. To characterize the transport and reactivity within the micro-heterogeneous clay rock matrix, we used a combination of synchrotron-based micro-X-ray fluorescence elemental imaging and micro-X-ray absorption spectroscopy. Two-dimensional reactive transport patterns and mappings of the geochemical heterogeneity were simultaneously recorded, complemented by the investigation of the local chemical speciation of Pu. The obtained chemical distribution patterns were subject of (phenomenological) reactive solute transport modelling considering diffusion and interfacial reactivity including sorption (surface complexation) as well as electron-transfer reactions.

## Results and discussion

**Figure 1:**
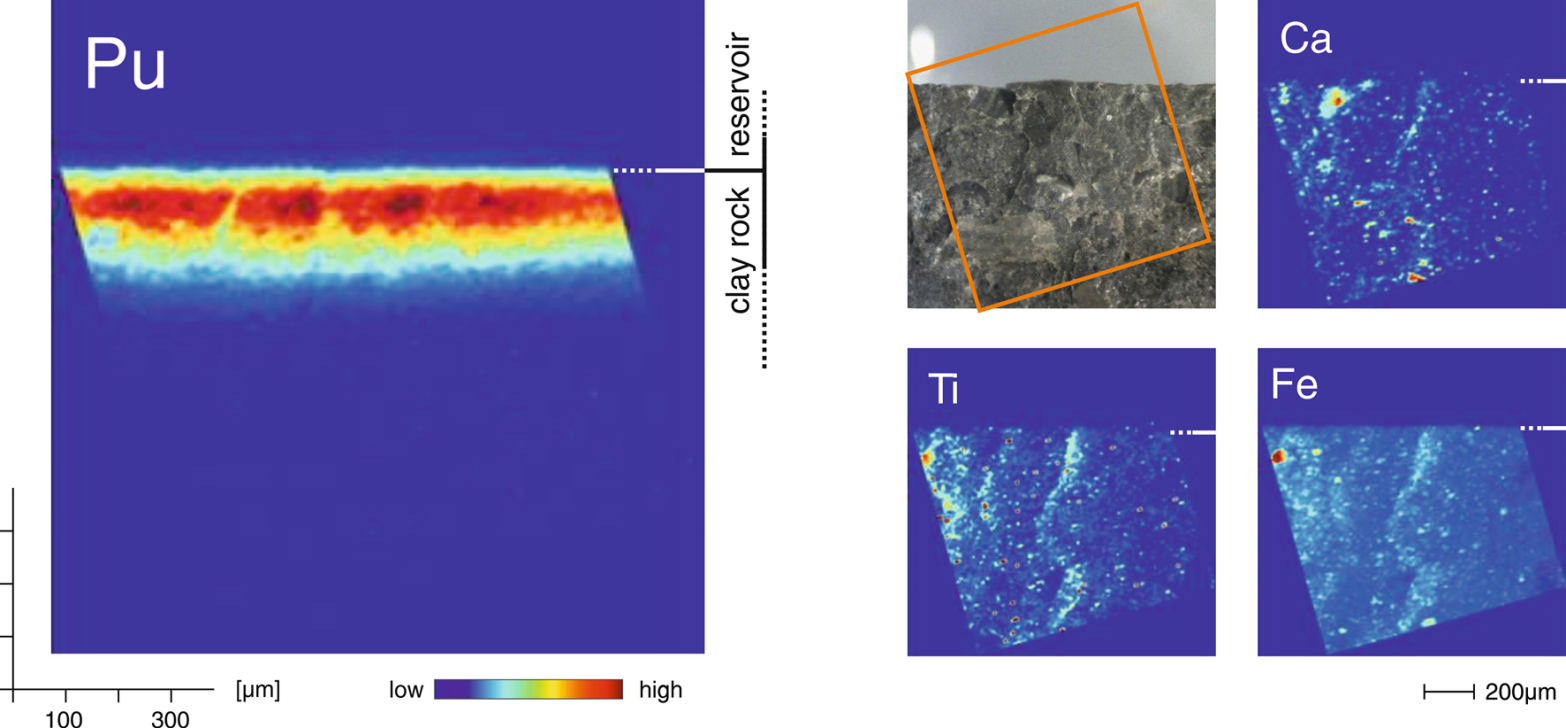
Chemical imaging of in-situ reactive transport pattern of Pu within the OPA rock. *Left panel* Reactive transport pattern of Pu represented by spatially resolved Pu concentrations, obtained by synchrotron-based µ-XRF. *Right panels* corresponding geochemical heterogeneity; shown are the distributions of the indicator elements Ca, Ti, and Fe as well as a light microscopy image.

Chemical images depicting the in-situ reactive transport pattern of Pu within the OPA rock volume as well as selected complementary geochemical information are shown in Fig. 1. The two-dimensional reactive transport pattern of Pu represented in the left panel of Fig. 1 is obtained based on spatially resolved measurements of Pu L_α_ fluorescence intensities. Accordingly, the chemical image illustrates the local variations of the total Pu concentration, independent of electronic state or chemical speciation. A definite local concentration gradient from the reservoir–clay rock interface into the rock is readily apparent. After the 26 days of diffusion, a migration distance of up to approximately 350 µm can be established (see also logarithmic representation of profile data given in Fig. 4). However, the observed reactive transport patterns do not correspond to a spatially uniform front as one would expect for diffusion in a homogeneous porous medium. A pronounced, characteristic ‘fingering’ is observed. This characteristic transport pattern results from local chemical (and physical) heterogeneities. Such local variations are potentially of high impact concerning the local mobility of Pu as its electronic state and reactivity are very sensitive to changes in local pH, Eh, and pore water composition. An illustration of the present local geochemical heterogeneity is given in the right-side panels of Fig. 1. Depicted are the distributions of the selected elements Ca, Ti, and Fe (complemented by a light microscopy image). While a rather explicit microscopic heterogeneity is observed, a distinct correlation between the measured elemental distributions and the observed Pu transport pattern is not immediately apparent. However, within particular local domains, clear impacts of heterogeneity on transport pattern can be observed. As an example, the prominent Fe-rich micro-domain shows a considerably reduced reactivity regarding the retardation of Pu (see Fig. S5 for corresponding zoom-in). The local Pu concentration within the Fe domain is clearly reduced and no enhanced reactivity is observed in the vicinity of the Fe-rich domain.

**Figure 2:**
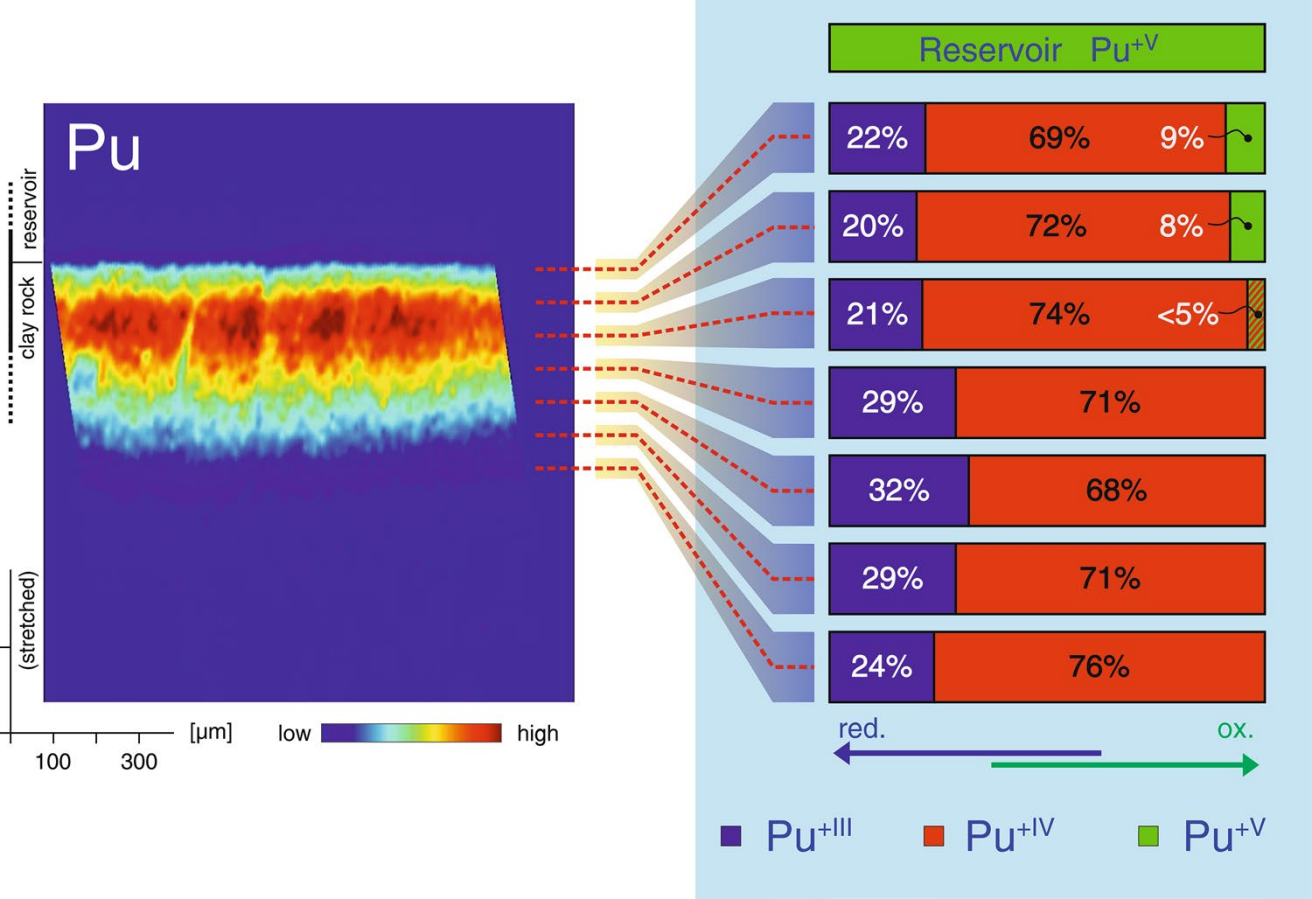
Distribution of Pu redox species across the diffusion profile. *Left panel* Reactive transport pattern of Pu represented by spatially resolved Pu concentrations, obtained by synchrotron-based µ-XRF. *Right panel* Fractions of Pu^+V^, Pu^+IV^, and Pu^+III^ respectively, observed at different locations along the direction of diffusion.

As the redox state of Pu corresponds to the key factor controlling the mobility of Pu in reactive porous media, potential redox state changes during the course of diffusion were investigated by means of spatially resolved Pu L_III_-edge XANES measurements. Starting near the reservoir–rock interface, µ-XANES spectra were recorded along the direction of diffusion every 50 µm, with a spatial resolution given by the beam size of about 2.5 µm. Information regarding the predominant redox states can be obtained by deconvoluting each µ-XANES spectrum by means of linear combination analysis (LCA) using known Pu reference XANES spectra. The corresponding results are shown in Fig. 2. The locations of the µ-XANES recordings are indicated with respect to the reactive transport pattern in the left panel (note the stretched representation along the direction of diffusion). The local distribution of oxidation states is illustrated on the right panel of Fig. 2. In the reservoir solution, Pu^+V^ is the predominant electronic state of plutonium (see also the corresponding Pourbaix Diagram given in SI, Fig. S2). In the rock, however, the distribution of Pu redox states is different. An obvious and immediate shift towards a larger fraction of more reduced states is observed. Already at the point closest to the reservoir–rock interface, the three redox states Pu^+V^, Pu^+IV^, and Pu^+III^ are simultaneously observed in the rock.

In its natural setting, Opalinus Clay corresponds to a strongly reducing environment. Indicative is the occurrence of pyrite and siderite within Opalinus Clay. The control of the local Eh is attributed to the sulfate/pyrite redox couple^35,36^. Typical Eh values reported in literature fall within the range of − 100 to − 300 mV (SHE). Despite an almost unavoidable exposure of the intact bulk rock material to oxygen during field sampling and sample preparation in the laboratory, a large fraction of the redox capacity of the rock material is preserved. Accordingly, a steep Eh gradient develops across the reservoir-rock interface.

**Figure 3:**
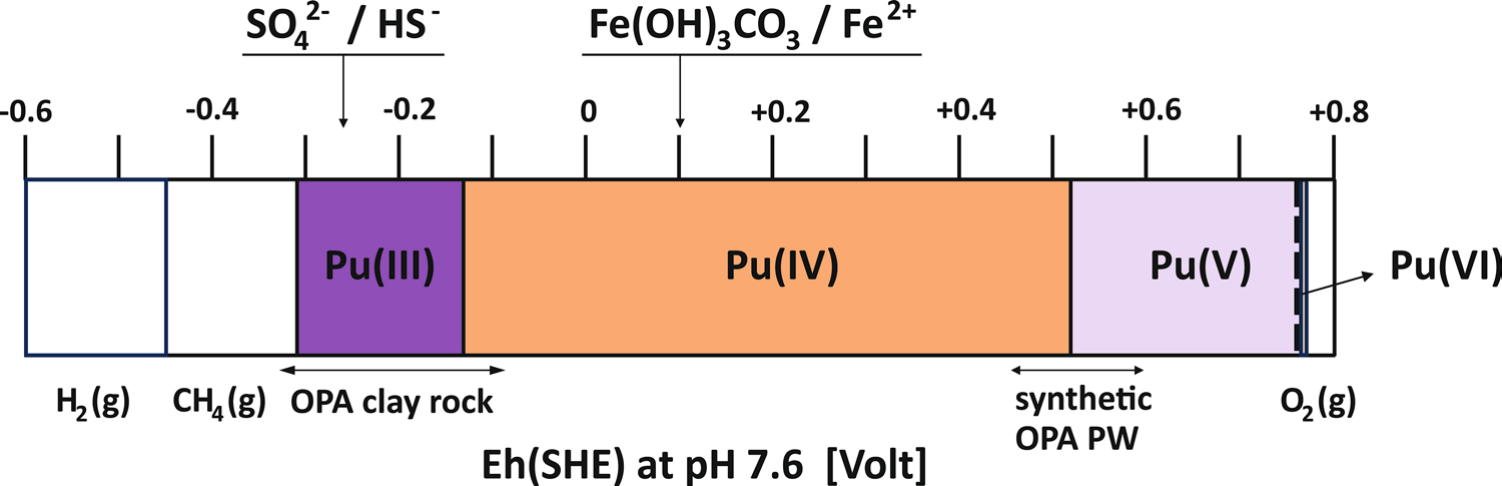
Graphical sketch summarizing the Eh conditions and corresponding Pu redox speciation relevant to the present reactive diffusion experiment.

A graphical sketch summarizing the Eh conditions and corresponding Pu redox speciation relevant to the present reactive diffusion experiment is depicted in Fig. 3.

Entering the pore space, interactions of Pu^+V^ with electron donors present in the clay rock lead to the immediate onset of reduction of Pu^+V^ and the corresponding build-up of Pu^+IV^ and Pu^+III^, with Pu^+IV^ being the predominant Pu species. With increasing migration distance into the rock, a trend towards a continual reduction of Pu is established. Pu^+V^ is observed only within the first ~ 100 µm from the interface, further downstream Pu^+V^ is below the detection limit. Towards the forefront of the diffusion front, only the states Pu^+IV^ and Pu^+III^ are observed. An illustrative example of a µ-XANES spectrum and related LCA is given in SI, Fig. S6. Depicted is the situation near the reservoir–rock interface. As shown, µ-XANES investigations yield information about the locally predominant electronic states of Pu (and potentially hints towards molecular speciation based on XANES ‘finger printing’^37^). In addition to near-edge analysis, μ-EXAFS measurements can provide supplementary fundamental details related to the molecular coordination of Pu along its migration path. Knowledge concerning the local chemical speciation of Pu within the barrier allows to identify and to differentiate between reaction mechanisms responsible for the retardation. Considering the demonstrated reduction of the Pu^+V^ to Pu^+IV^ (and Pu^+III^), precipitation of Pu^+IV^ phases with low solubility has to be considered in addition to the ‘classical’ types of adsorption reactions. Furthermore, information related to the potential formation of polymeric surface complexes^38^ as well as polynuclear Pu species or colloidal Pu^39–42^ is transport relevant.

Two examples of obtained μ-EXAFS spectra recorded with a 2.5 µm beam are shown in SI-Fig. S7 (left panel). The two spectra correspond to a comparison of two domains of the determined reactive transport pattern. Note, the μ-EXAFS spectra were collected with a spatial displacement (parallel to the reservoir–rock interface plane) compared to the series of XANES data shown in Figs. 2 and S6. A first spectrum was collected at the peak concentration within the diffusion front, roughly 100 µm from the interface. A second spectrum reveals speciation information from the forefront of the diffusion front. Due to the low local Pu concentration and the restricted acquisition time, the counting statistics is limited. The accessible k-range is restrained to a maximum of ~ 8 Å^−1^. Due to the resulting shell resolution limit of ΔR of 0.26 Å, the analysis is at first limited to the first shell. Several spectroscopic and theoretical studies revealed characteristic Pu–O distances for the aqueous Pu species Pu^+III^, Pu^+IV^, and Pu^+V^^37,43–45^. Relevant to the present case, the reduced Pu species Pu^+III^ and Pu^+IV^ have a uniform, spherical first shell distribution of eight to nine oxygens at 2.49 ± 0.03 Å and at 2.35 ± 0.04 Å, respectively. The corresponding Debye–Waller factors are approximately 0.007–0.012 Å^2^^25,45,46^. Discriminative, Pu^+V^ coordination is characterized by two axial oxygen atoms, O_ax_ located at 1.82 ± 0.03 Å and 4–5 equatorial oxygen atoms at a distance of 2.47 ± 0.03 Å from the central Pu. Observed Debye–Waller factors of the axial and equatorial oxygen shells of the plutonyl moiety are approximately 0.002 Å^2^ and 0.005–0.008 Å^2^, respectively^44^. The spectroscopic observation of a short Pu–O distance of ~ 1.82 Å is therefore indicative of the presence of Pu^+V^. Based on the present data (with an expected shell resolution limit of ΔR ≈ 0.26 Å), the difference in Pu–O distance of axial Pu^+V^–O_ax_ pairs as compared to the Pu–O distance of the reduced Pu forms can be resolved by EXAFS analysis. For both spectra, the first shell can be fitted satisfactorily by a single Pu–O scattering pair with a distance of roughly 2.31 Å. However, the enhanced value of the Debye–Waller factor of approximately 0.02 Å^2^ indicates a broader distribution of Pu–O distances. These results are indicative of the predominant presence of a mixture of reduced Pu species Pu^+IV^ and Pu^+III^. This conclusion is consistent with the analysis of the µ-XANES measurements along the diffusion path (Fig. 2 and SI-Fig. S7), and confirms the considerable reduction of Pu^+V^ during migration into the clay rock.

To elaborate further on the validation of the in-situ observation of Pu reduction, an anaerobe batch sorption study has been conducted (see SI for further details). During each step of this specific experiment, every reasonable endeavor has been made to limit the contact with oxygen. As a result of these efforts, the final Eh of the Opalinus Clay rock–pore water suspension was -59 mV (SHE), remaining close to the in-situ Eh condition of Opalinus Clay rock settings (− 100 to − 300 mV (SHE)). XANES and EXAFS results (shown in SI, Fig. S7, right panel) document the occurrence of Pu^+III^ as the result of redox reactions involving components of the OPA material acting as electron donors.

The capability to record spatially resolved spectroscopic information at characteristic locations of the migration pattern yields mechanistic, transport relevant information. For the studied case, as basic process, the more mobile Pu^+V^ present in the reservoir is diffusing into the rock. Immediately after entering the clay rock material, the Pu^+V^ is involved in electron-transfer reactions. Products of these redox reactions are the less mobile, reduced forms Pu^+IV^ and Pu^+III^. Simultaneously, all three Pu species are subject to complexation or chemical bonding to reactive surface sites resulting in an up-concentrating of the three Pu species at solid–liquid interfaces of various minerals. This reaction scheme is deduced from the simultaneous presence of all three Pu species near the reservoir as evidenced by µ-XANES. The reduced forms Pu^+IV^ and Pu^+III^ are expected to show an apparent diffusivity diminished by as much as 2–3 orders of magnitude compared to Pu^+V^^29,47^. Consequently, the reduction of Pu^+V^ within the Opalinus Clay rock leads to a significant decrease in the spreading velocity of Pu in the rock. In this context, the observed Pu speciation at the forefront of the diffusion profile is quite striking. Only the reduced forms Pu^+IV^ and Pu^+III^ with high retardation are present, the considerably more mobile Pu^+V^ is absent (Fig. 2). This observed pattern is at first counterintuitive as Pu^+V^ with the lowest affinity for the reactive surface sites of the clay rock (lowest ‘distribution coefficient K_D_’) can be expected to migrate the fastest into the clay rock. Accordingly, a gradually higher fraction of Pu^+V^ is anticipated towards the forefront of the diffusion while a reverse tendency is observed experimentally.

**Figure 4:**
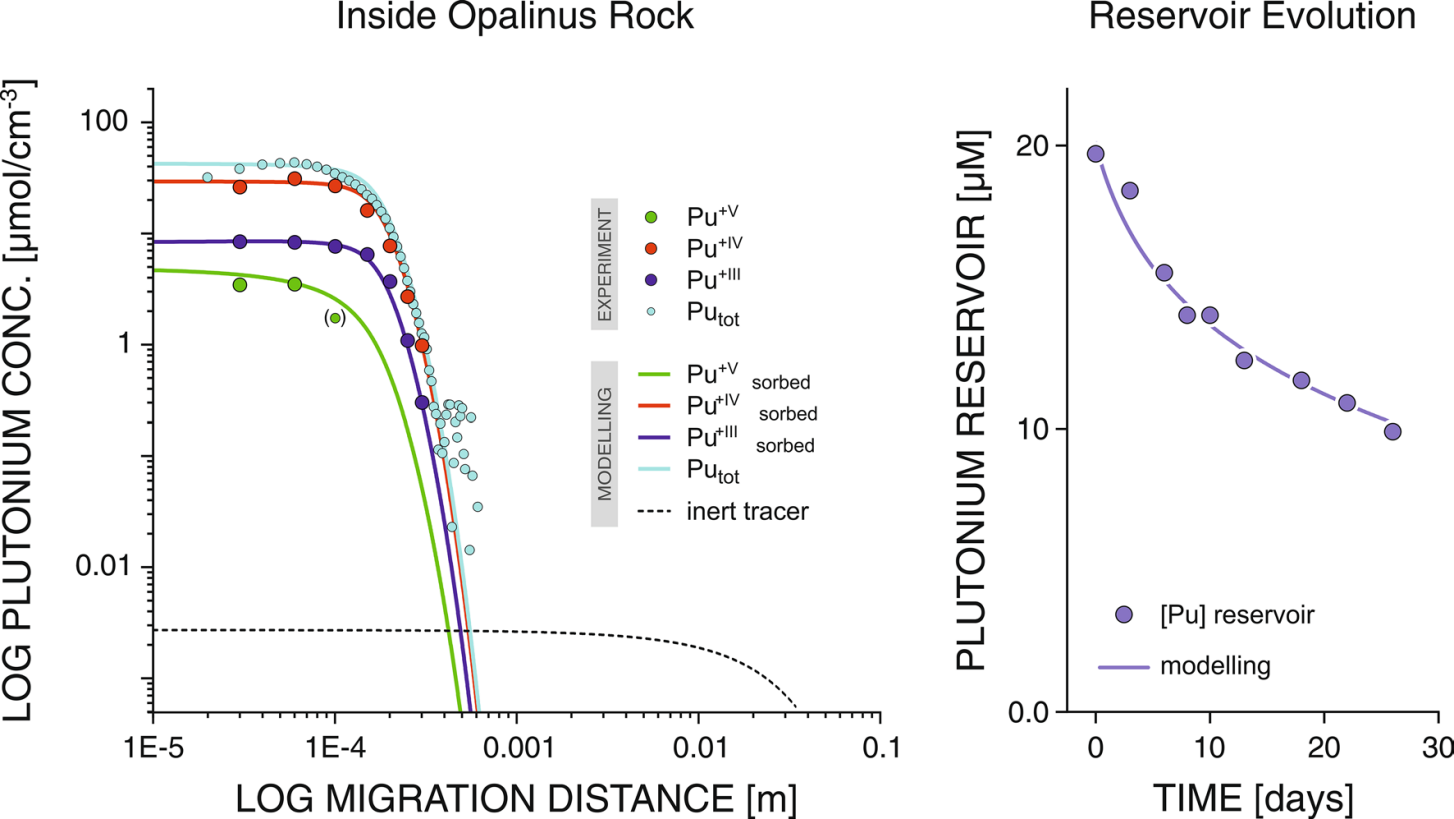
Diffusion profiles of Pu species in Opalinus Clay. *Left panel* Profile for total Pu concentration as well as the individual profiles for Pu^+V^, Pu^+IV^, and Pu^+III^ in a double logarithmic representation. *Right panel* Evolution of the Pu concentration in the reservoir over the course of the diffusion experiment. Both panels include the results of phenomenological modelling (lines).

This remarkable migration behaviour of the different redox species is further emphasized by inspecting and comparing the diffusion profiles of the individual Pu species. The integrated diffusion profiles obtained from the two-dimensional Pu transport pattern (Fig. 1, left panel) are depicted in Fig. 4 (left) in a double logarithmic representation. Shown are the profile for the total Pu concentration as well as the individual profiles for the different Pu oxidation states. Despite their expected 2–3 order of magnitude higher retardation, Pu^+IV^ and Pu^+III^ are penetrating deeper into the clay rock as compared to Pu^+V^.

Furthermore, examining the shape of the diffusion profile reveals two particular features deviating from the reactive transport pattern predicted based on ‘classical’ diffusion and retardation based on linear sorption. The observed shape recalls the characteristics of a self-sharpening front. First, within the first approximately 150 µm a rather constant Pu concentration is established. This plateau is followed by a concentration decrease with an exceedingly high gradient.

Phenomenological modelling is used to rationalize the observed migration behaviour. The outcome of a satisfying model realization is included in Fig. 4. As relevant mass balance constraint, the evolution of the Pu concentration in the reservoir over the course of the migration experiment (Fig. 4, right) is modelled together with the rock diffusion profiles.

Two different phenomena could be identified, which potentially contribute to the observed peculiarities of the Pu migration. First, pronounced differences in the characteristics of the sorption isotherm of Pu^+V^ as compared to the adsorption behaviour of Pu^+IV^ and Pu^+III^ represent a potential rationale. For the case of a highly non-linear sorption isotherm of Pu^+V^ and nearly linear isotherms for Pu^+IV^ and Pu^+III^, the pronounced self-sharpening effect due to the isotherm non-linearity would lead to a reduced apparent dispersion of Pu^+V^ at the forefront of migration. Indeed, there is evidence for nearly linear sorption isotherms for Pu^+IV^ at low concentrations, e.g., sorbing to the reactive barrier material bentonite^30^. Further, a considerable non-linearity of the sorption isotherms in case of Pu^+V^ for elevated concentrations (> 10^–8^ mol/L) was reported^48^. A dependence of the K_D_ on the solution concentration would mainly lead to an increased steepness of the concentration gradient at the diffusion forefront.

Considering the coupled redox reactions leads to a second mechanism. In a complex material such as OPA, a variety of electron donor/acceptor pools are possibly present. The observed peculiarities concerning the distribution of the different redox species as well as regarding the shape of the diffusion profile can be rationalized by the presence of a highly reactive electron donor pool, but present only in small quantities resulting in a limited, finite redox capacity. All Pu^+V^ entering the rock material is reduced at fast rates by this highly reactive electron donor component. While Pu^+V^ is vanishing locally, Pu^+IV^ and Pu^+III^ are produced. Pu^+V^ will not be able to progress further into the rock, until the redox capacity of the highly reactive electron donor pool is entirely depleted locally. Contrary, the produced Pu^+IV^ and Pu^+III^ are able to migrate into the rock, obviously with considerable retardation. As a result, the reduced Pu species appear to move faster as compared to the more oxidized Pu^+V^. The phenomenological modelling included in Fig. 4 is based on such a mechanistic scenario, including a depleting trace pool of a highly reactive electron donor.

The diffusion front has been imaged over an extended field of view of more than 4 mm (of which a representative section is shown in Fig. 1). After reduction to Pu^+IV^ and Pu^+III^, the mobility of Pu will be considerably limited. As the larger fraction of Pu is in reduced forms, the distribution of Pu corresponds to a mapping of the local electron door concentrations. No radial gradients are observed, the Pu diffusion appears as a band-like pattern over the entire range investigated (see SI, Fig. S5). Accordingly, the electron donors responsible for the reduction of Pu^+V^ have to be distributed finely dispersed within the rock material.

The proposed concomitance of multiple electron donor pools with different reactivities (kinetics), capacities, and spatial distribution characteristics is in accordance with results elaborated by Howing et al.^49^ for the case of Boom Clay. Based on mediated electrochemical oxidation and reduction measurements, their study revealed structural Fe^2+^ to be a finite, highly reactive, spatially dispersed electron donor pool, while pyrite and siderite represent localized redox-active inventories with higher capacities but slower kinetics.

The sample revealing the Pu reactive transport pattern (Fig. 1) was obtained by cleaving, which preferentially occurs along the bedding planes within the clay domains of the clay rock. Consequently, the observed geochemical heterogeneity represents the variation within these clay domains. However, the used clay rock material includes additional (hierarchical) heterogeneities. To be noted, the used OPA material was extracted from a shaly facies^50^ which includes considerable geochemical heterogeneities on various length scales, e.g., characteristic CaCO_3_ domains. An example of such meso-scale geochemical variability was revealed by investigating the rock surface exposed to the reservoir. This experimental rock surface is the result of cutting bulk rock material by sawing without preference for expressing a particular domain.

**Figure 5:**
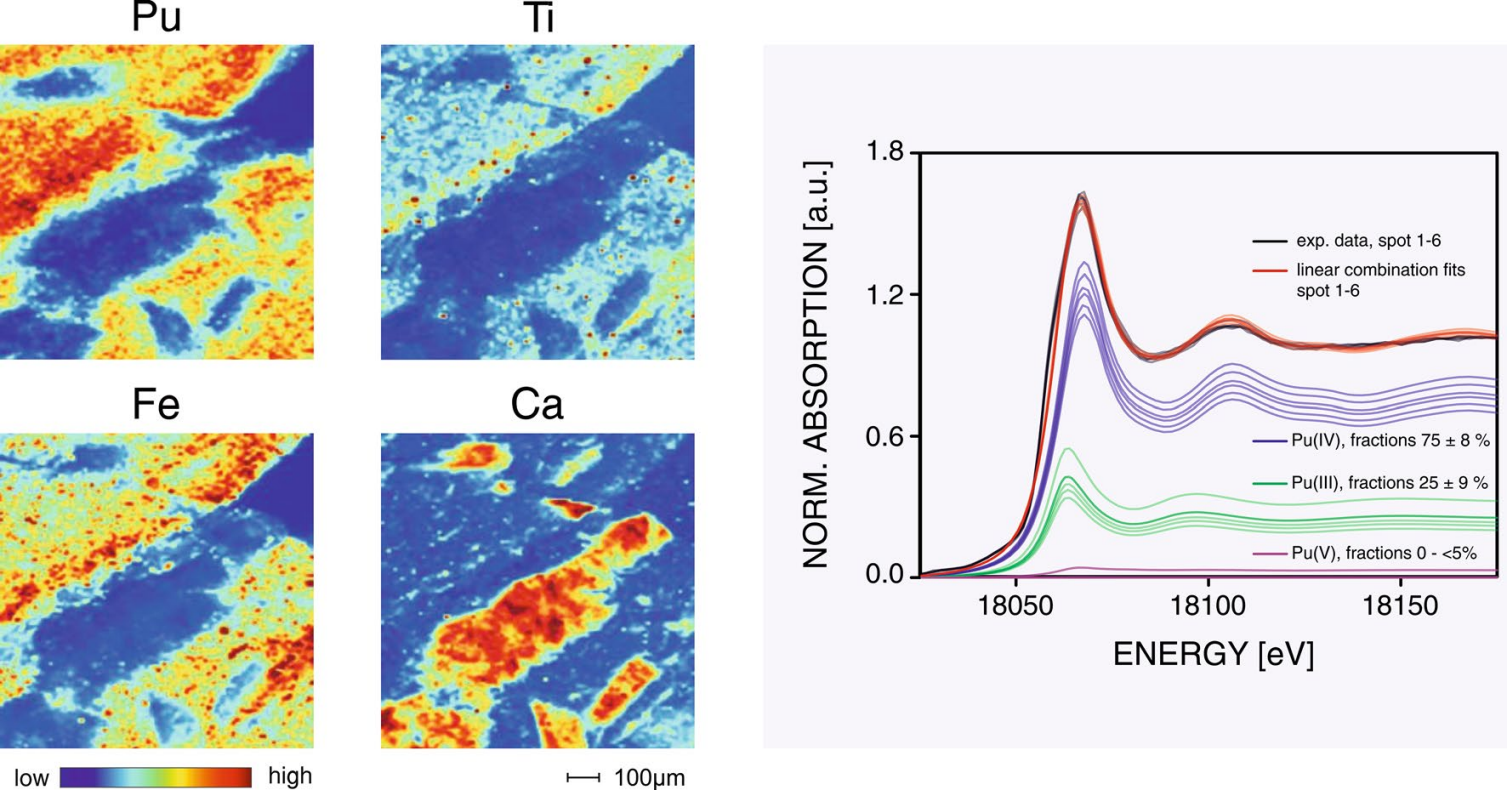
Impact of geochemical heterogeneity on reactive transport pattern. *Left panel* µ-XRF mappings showing the spatial correlation between the distributions of Pu and the geochemical indicator elements Ca, Fe, and Ti. *Right panel* Multiple Pu L_III_-edge µ-XANES spectra recorded within domains revealing elevated Pu retention. Experimental data are shown together with the corresponding LCA results.

Figure 5 shows the spatial distribution of Pu within the rock surface exposed to the reservoir. Reactive domains exhibiting high local Pu concentrations are entangled by inert domains with no Pu present. Typical length scales of the spatial variations are in the order of hundred(s) of micrometers. The areas of high and low Pu concentrations clearly correlate with the geochemical characteristics of the rock material. This is readily apparent by comparing the local Pu concentration with the geochemical indicator elements Ca, Ti, and Fe (Fig. 5). In the present case, Ca is indicative for CaCO_3_ domains, while clay rich domains yield elevated Ti and Fe concentrations. Comparing the distribution of Ca and Pu in the μ-XRF mapping, it becomes readily evident that Pu has no affinity towards the CaCO_3_ phase. The calcite-rich domains do not contribute to the retention of Pu. Opposite, in the clay domains, outlined by the indicator elements Fe or Ti, an enrichment of Pu is observed. This set of chemical images depicting expressed reactive transport pattern in direct relation with the geochemical heterogeneity corresponds to an illustrative example concerning the decisive role of the geochemical heterogeneity in the context of Pu migration through a reactive barrier. The different compositional domains of the OPA material result in pronounced gradients of the local Pu concentrations and the reactive transport pattern appear to be fragmentary. Clay rocks represent hierarchical materials characterized by various geochemical heterogeneities with distinctive length scales from nm to meters. To assess the potential impact of these heterogeneities, the analysis of reactive transport patterns within intact rock samples are essential.

The right panel of Fig. 5 shows several normalized Pu L_III_-edge XANES spectra. These spectra were obtained from multiple locations with elevated local Pu concentrations (see marked spots in SI-Fig. S8). The outcome of the corresponding LCAs is included in the figure. At the boundary to the reservoir, the measured Pu speciation shows only moderate variability and is consistent with Pu speciation measured near the inlet in the diffusion profile (SI-Fig. S6, left panel).

## Conclusions and implications

The present study expounds fundamental environmental processes relevant to the spreading of plutonium in a geological reactive barrier under conditions relevant to deep geological waste storage. The study is based on the innovative concept of chemical imaging of undisturbed reactive transport patterns expressed within the reactive porous medium. By this approach, detailed spatial distributions (‘images’) of the local Pu concentration are complemented by spatially resolved chemical speciation information (electronic state, molecular coordination) as well as corresponding geochemical information. Unique information regarding relevant processes is obtained, information which cannot be elaborated by alternative approaches in a straightforward manner.

The present investigations document—by direct experimental evidence—the decisive role of coupled redox and adsorption processes in the context of Pu mobility. The obtained data provide direct proof of reduction reactions occurring during the spreading of Pu in the geological (barrier) material. Along its migration path, Pu^+V^ is progressively reduced to Pu^+IV^ and Pu^+III^. The characteristic evolution of the fractions of Pu redox species along the direction of migration into the rock provides strong evidence towards a fundamental role of different electron donor pools present in the barrier. Specifically, the systematic distribution of different redox species and the peculiar shape of the diffusion profile, both point towards the existence of a highly reactive electron donor pool with limited capacity complemented by an addition fraction of electron donors with slower reaction rates but larger redox capacity.

In principle, Pu^+V^ is the mobility determining Pu species, however the effective migration velocity is controlled by the reaction rates of the reduction to Pu^+IV^ and Pu^+III^ and the redox capacity of the involved electron donor pools of the porous medium. Generally, hitherto only limited scientific information is available concerning the geochemical identity, buffering capacities, and reactivity (reaction rates) of redox-active components present in reactive barriers. Chemical imaging of reactive transport pattern has considerable potential to contribute to fill this crucial knowledge gap. Further, the present study documents the necessity of investigations on intact, undisturbed systems. Environmentally relevant materials are commonly heterogeneous and chemically highly complex structures, accordingly expressing complex reactivity. Imaging the chemical reactivity of intact systems is key to identify reactive components, reaction pathways, and coupling of reactions. Reactive (solute) transport pattern expressed in reactive porous media are very sensitive to underlying coupled reactions and corresponding kinetic rates. Time and space evolution results in the development of characteristic domains, fronts, and/or gradients. Analyzing the spatial context of local reactive transport pattern, ideally species specific, is at first essential to decipher and/or confirm the fundamental reactive processes occurring in natural and engineered repository barriers. Consequently, the visualization of reactive transport patterns by chemical imaging provides unique and rare datasets to substantiate proposed geochemical mechanisms and corresponding reaction rate laws and to validate reactive transport models and their corresponding predictive capabilities.

Specifically, the present study provides new insights into the complex interactions between Pu and the geological environment, in the present case the micro-heterogeneous clay rock matrix. Experimental results and modeling emphasize the dominant role of electron transfer reactions determining the mobility of Pu in reactive barrier systems. To improve our predictive capabilities further, an improved understanding of the nature and capacity of different electron donor–acceptor pools present in the reactive barrier material is fundamental.

The obtained information represents an essential contribution in evaluating the long-term safety of possible repository concepts and increases the confidence in clay rock as a host medium for the geological disposal of radioactive waste. The findings contribute to the development of advanced and effective geological nuclear waste disposal strategies.

## Materials and methods

### Reactive barrier material and Pu^+V^ reservoir solution

Details regarding the employed Opalinus Clay rock material and the preparation of the ^242^Pu^+V^ stock solution can be found in the Supporting Information (SI).

### Preparation of in-situ diffusion sample

As sample for the diffusion experiment, a clay cylinder was cut out of an OPA bore core with occasional exposure to ambient atmosphere. The bore core was obtained from the Mont Terri Underground Rock Laboratory^51^. Prior to generating the subsample, the orientation of the bedding planes in the main core was determined. The direction of the volume sampling was aligned to obtain an orientation of the clay rock layering perpendicular to the final sample surfaces. This subsample was stabilized by embedding in an epoxy resin (MEKP Härter, Fiberglas Discount, Germany). The embedded, pre-orientated clay rock volume was installed in the diffusion cell such that the diffusion proceeded parallel to the bedding planes. The employed diffusion cell corresponds to a modified version of the setup described by Van Loon et al.^52^. As modification, the cell was operated without steel filters to avoid chemical interaction of Pu with the steel filters. After mounting, the sample was preconditioned with synthetic OPA porewater (pH 7.6, total ionic strength of I = 0.4 M, Eh =  + 450 mV (SHE); see SI)^35,36^. In order to limit the spectral interferences between strontium (Sr) and Pu during the X-ray fluorescence based chemical imaging (see below), the synthetic pore water was prepared without the addition of Sr. In order to avoid bacterial growth, 0.003 M NaN_3_ was added to the pore water. The pre-equilibration was conducted for four weeks at 20 ± 2 °C. The synthetic pore water was continuously circulated between the reservoir and the rock interface by means of peristaltic pumping. After equilibration, the synthetic OPA pore water was replaced by a ^242^Pu^+V^ spiked (2 · 10^–5^ M) pore water solution (see SI). The Pu containing reservoir solution was in contact with the OPA clay for 26 days during which the reservoir was continuously mixed by recirculating flow. In order to document the evolution of the ^242^Pu^+V^ concentration in the reservoir, 0.1 mL aliquots of reservoir solution were sampled every second or third day. Based on these aliquots, the remaining ^242^Pu^+V^ concentration in the reservoir was measured using a self-made liquid scintillation counting (LSC) setup.

At the end of the diffusion experiment, the clay core was removed from the cell and excess solution removed by wiping and final evaporation. To access the reactive transport expressed in the interior of the sample, the rock sample was cleaved into two halves (cleaving takes place preferably along the bedding planes of the OPA). As preparation for the synchrotron microprobe measurements, two subsamples were obtained by cutting off excess material, oriented and fixed in a Plexiglas holder. One sample was mounted providing view onto the surface that had been in contact with the solution (reservoir–rock interface), the second sample was prepared exposing the freshly cleaved surface parallel to the diffusion direction. Illustrations of the experimental workflow are given in SI, Figs. S3 and S4.

### Synchrotron-based chemical imaging and spatially resolved spectroscopy

Synchrotron-based measurements have been performed at the the microXAS beamline (X05LA) of the Swiss Light Source (SLS). The SLS represents a 2.5 GeV storage ring routinely operating in top-up mode at 400 mA current. At the microXAS microprobe facility undulator radiation is dynamically focused by means of a Kirkpatrick-Baez reflective mirror system, in the present case down to a final spot size of ~ 2.5 × 1 µm^2^ (hor × vert). Monochromatic radiation was produced by a fixed-exit double-crystal monochromator using the option of the Si crystal pair with (111) orientation. The monochromator was calibrated by measuring the absorption edge of a metallic Zr foil. The first inflection point was set to 17,998 eV. To record chemical images based on micro-X-ray fluorescence (µ-XRF), an energy of the incident beam of 18,067 eV was used to optimize the response of the Pu signal. Fluorescence radiation was recorded using a single-element silicon drift detector (SDD; KETEK GmbH, Germany).

To eliminate the spectral interferences between geogenic Sr (K_α1_ at 14,165 eV; K_α2_ at 14,098 eV) and the low experimental concentrations of Pu (L_α1_ at 14,279 eV; L_α2_ at 14,084 eV), the edge-difference methodology was employed to obtain images of net intensity for Pu. Accordingly, to elaborate a chemical image of Pu, two XRF maps were recorded, one at 18,067 eV (above the Pu L_III_ edge) and a second one at 18,000 eV (below Pu L_III_ edge).

Spatially resolved Pu L_III_-edge X-ray absorption near-edge spectroscopy (μ-XANES) measurements were recorded in fluorescence mode. Background correction and normalization of the spectra were performed using the software package Athena^53^. The fractions of different Pu oxidation states present were determined by linear combination fitting (using the sub-program provided by the Athena package). The experimental μ-XANES spectra were fitted by a combination of standard spectra of Pu^+III^/Pu^+IV^/Pu^+V^/Pu^+VI^^44,46,54,55^ over the energy range of 18,035–18,155 eV.

Due to moderate count rates (Pu was present in low concentrations), the collection of spatially resolved extended X-ray absorption fine structure (µ-EXAFS) spectroscopic data was limited to a k-range up to ~ 8 Å^−1^. Corresponding data processing and spectral analysis were performed with the programs Athena^53^ and EXAFSPAK^56^. Theoretical scattering phases and amplitudes were calculated with FEFF9.6^57^ using the crystal structures of calcium-silicate-hydrate^58^, where incorporated U was substituted by Pu.

### Reactive solute diffusion modelling

A phenomenological model of minimal complexity was constructed (see also the conceptual model approach by Fjeld et al.^59^). The transport of dissolved Pu species has been modeled by means of a classical dispersion model coupled to chemical reactions. Three different aqueous Pu species were considered (Pu^+V^, Pu^+IV^, Pu^+III^). The motion of mobile aqueous species in the pore water is coupled to reactions involving immobile species. The considered chemical reactions involve two types of interfacial reactions. First, ‘sorption’ reactions between aqueous species present in the pore solution and reactive surface sites of the immobile solid matrix. Three different reactive sites are used, one specific for each Pu redox state. Generic Langmuir adsorption isotherms are utilized to formulate the mass action laws. The chemical equilibria are modeled by elementary kinetic rate laws for the forward and backward reactions. The second type of chemical reaction represents the surface mediated reduction of Pu. Two different pools of electron donors are considered to mimick the expected geochemical multiplicity. The geochemical reactive (solute) transport model is summarized in SI-Table S3. Concerning the computational implementation, the resulting set of coupled partial differential equations is solved with existing numerical algorithms (Numerical Algorithms Group, 1996). The constructed reactive transport code was extensively tested against analytical solutions available for several special cases such as inert tracer or linear sorption (see e.g., Jury and Roth^60^). An analogous model implementation was employed to describe reactive solute transport in porous media involving mobile colloidal particles^61,62^.

Initial guesses for rock porosity, reactive surface site density, effective diffusion coefficient (D_e_) as well as sorption affinities were based on literature values (SI-Tables S3 and S4). Optimized values for porosity and D_e_, specific surface site concentrations, electron donor pool capacities as well as corresponding reaction rates for both types of reactions were obtained by iterative matching of simulation results to both, the experimentally observed one-dimensional diffusion profiles for the different Pu redox species and the corresponding temporal evolution of the reservoir concentration.

### Supplementary Information

Supplementary Information.

## Data Availability

The datasets produced and analysed during the current study are available from one of the corresponding authors on reasonable request.
