# Supplementary material for: Plutonium mobility and reactivity in a heterogeneous clay rock barrier accented by synchrotron-based microscopic chemical imaging
Source: Sci Rep. 2024 Feb 7;14:3087. doi: 10.1038/s41598-024-53189-8 (PMC10847135; doi:10.1038/s41598-024-53189-8)
Supplement: Supplementary file 1 — Supplementary Information. [file 41598_2024_53189_MOESM1_ESM.pdf]

Plutonium Mobility and Reactivity in a Heterogeneous Clay Rock Barrier Accented by Synchrotron-based Microscopic Chemical Imaging

U. Kaplan[1], S. Amayri[1], J. Drebert[1], D. Grolimund[2]✉, T. Reich[1]✉

[1] Johannes Gutenberg-Universität Mainz, Department of Chemistry, 55099 Mainz, Germany

[2] Swiss Light Source, Paul Scherrer-Institut, 5232 Villigen PSI, Switzerland

✉ Corresponding authors:
treich@uni-mainz.de (+49 6131 3925250); daniel.grolimund@psi.ch (+41 56 310 4782)

Number of pages: 16

Number of tables: 4

Number of figures: 8

1. Supplementary information related to Materials and Methods

1.1 Opalinus Clay Rock Material

Opalinus Clay (OPA) rock material was used as a realistic representative of a reactive barrier to be used in a future nuclear waste repository. In this project subsamples from the core BLT14[1] from the rock laboratory (Felslabor) Mont Terri[2,3], Switzerland, was employed. Core BLT14 corresponds to a shaly facies. The main mineral components and elemental composition of the employed OPA material were identified by laboratory-based X-ray diffraction (XRD) and fluorescence (XRF)[4]. The present OPA sample consists of several clay minerals (including 22 ± 2 % kaolinite, 23 ± 2 % illite, 11 ± 2 % illite/smectite and 10 ± 2% chlorite), 14 ± 4% quartz, 13 ± 8 % calcite, and 4.1 ± 2.3 % Fe^{+II}-bearing minerals such as pyrite and siderite. Moreover, OPA contains small amounts of albit (~1 %), K-feldspars (~1 %), and organic carbon (~0.8 %). Continuative information on relevant physical-chemical properties of OPA can be found in ref[5].

1.2 Opalinus Clay Rock Pore Water

Synthetic OPA pore water was used as background electrolyte during pre-equilibration and the diffusion experiment. The synthetic OPA pore water has a pH of 7.6 and an ionic strength of 0.4 M. It consists of $CaCl_2$, $MgCl_2$, KCl, $SrCl_2$, Na_2SO_4, and $NaHCO_3$ [6,7]. All solutions were prepared using Milli-Q water (18.2 $M\Omega$ cm Synergy™ Millipore water system, Millipore GmbH, Schwalbach, Germany) and chemicals used were from p.a. (pro analysis) quality grade.

In order to limit the spectral interferences between Sr and Pu during the X-ray fluorescence based chemical imaging (Sr: $K_{\alpha1}$ at 14’165 eV; $K_{\alpha2}$ at 14’098 eV; Pu: $L_{\alpha1}$ at 14’279 eV; $L_{\alpha2}$ at 14’084 eV), the synthetic pore water was prepared without the addition of Sr. In order to avoid bacterial growth, 3 mM NaN_3 was added to the pore water. The exact composition of the synthetic pore water is given in Table S1.

Table S1:

Composition of the synthetic Opalinus Clay pore water.[6,7]

component	mmol/L
Na^{+}	240
K^{+}	1.6
Mg^{2+}	16.9
Ca^{2+}	25.8
[Sr^{2+}]#	*[0.5]*
Cl^{-}	300
SO_4^{2-}	14.1
CO_3^{2-}	0.5
NaN_3	3.0*

# To limit spectral interferences between emitted Sr and Pu X-ray fluorescence during chemical imaging, the employed synthetic pore water was prepared without the addition of Sr.

* NaN_3 was added to prevent bacterial growth during the diffusion experiment.

1.3 ^{242}Pu Stock Solution

The diffusion experiment was carried out with ^{242}Pu in aqueous solution. ^{242}Pu solution was evaporated to dryness and the residue was dissolved in 10 M HCl (with some drops of conc. HNO_3 to oxidize all Pu to Pu^{+VI}). This $^{242}Pu^{+VI}$ stock solution was purified from its decay products using anion exchange chromatography at 55 °C (4 mm diameter, 150 mm length glass column filled with Dowex AG 1-X8 purchased from Bio-Rad Laboratories, Hercules, USA). After a washing step with 8 M HCl, Pu was eluted with 0.5 M HCl. The eluate was evaporated to dryness, fumed three times with 1 M $HClO_4$ (not to dryness), and dissolved in 1 M $HClO_4$. Subsequently, the pH was adjusted using diluted NH_3. The final Pu^{+V} stock solution was obtained from the purified Pu^{+VI} stock solution by potentiostatic electrolysis at pH 2.5. The oxidation state purity was verified by UV/Vis spectroscopy at the characteristic absorption bands at 568 nm for Pu^{+V} [8] (see Fig. S1). The Pu concentration in the stock solutions was determined by liquid scintillation counting (in-house) using the scintillation cocktail Ultima Gold XR (Perkin Elmer, USA) and equaled $1.4 \cdot 10^{-4}$ M for ^{242}Pu. All solutions were prepared using Milli-Q water (18.2 MΩ cm Synergy™ Millipore water system, Millipore GmbH, Schwalbach, Germany) and chemicals used were from p.a. (pro analysis) quality grade.

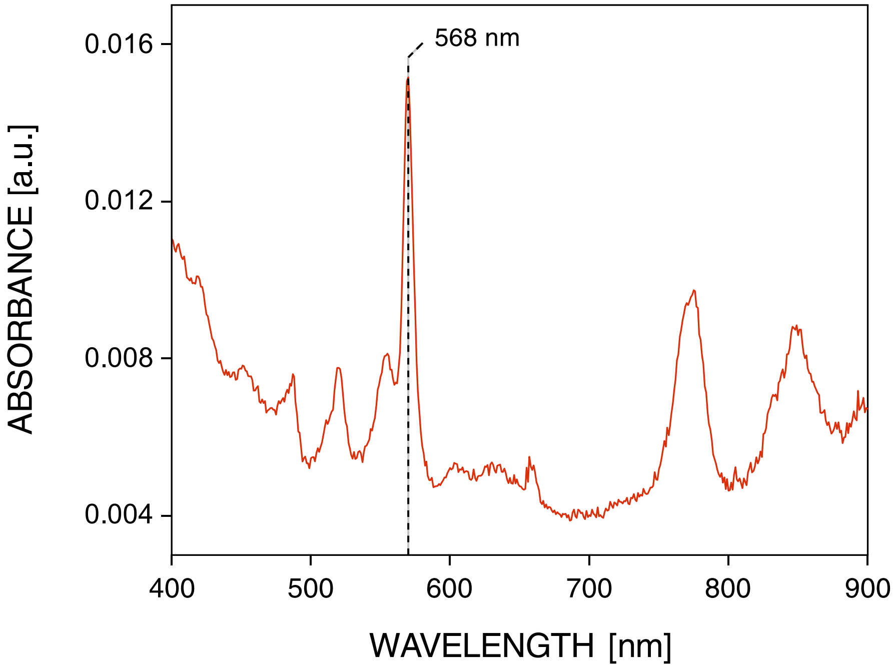

Figure S1: UV/Vis spectrum of the $1.4 \cdot 10^{-4}$ M Pu stock solution at pH 2.5 confirming the predominant presence of Pu^{+V}.

1.4 ^{242}Pu Experimental Solution (Reservoir)

A given aliquot of Pu^{+V} stock solution was added to OPA pore water at pH 7.6 to obtain a final concentration of 20 µM Pu. The measured Eh value in the primary reservoir (V = 110 mL) in contact with the diffusion sample was +450 (±50) mV (SHE). In freshly prepared OPA pore water, the corresponding Eh value was somewhat higher, e.g., +600 (±50) mV (SHE). For Eh = +600 mV, the predominant Pu species present in the pore water can be expected to be Pu^{+V} [9] (see Fig. S2). Independent measurements of Pu in OPA pore water employing CE-ICP-MS showed that Pu^{+V} is the only species present in solution [10,11]. As the calculation in Fig. S2 shows, at Eh = + 450 mV, one could also expect the presence of Pu^{+IV} in solution. However, at pH 7.6 this Eh value is at the border between the predominance of PuO_2^+ and $Pu(OH)_{4(aq)}$. Therefore, the presence of Pu^{+IV} in the OPA pore water during the diffusion experiment cannot completely be ruled out. However, the Pu concentration in the primary reservoir changed only from 20 µM to 10 µM during 26 days (see Fig. 4, right panel), indicating that no precipitation of $Pu(OH)_4$ occurred.

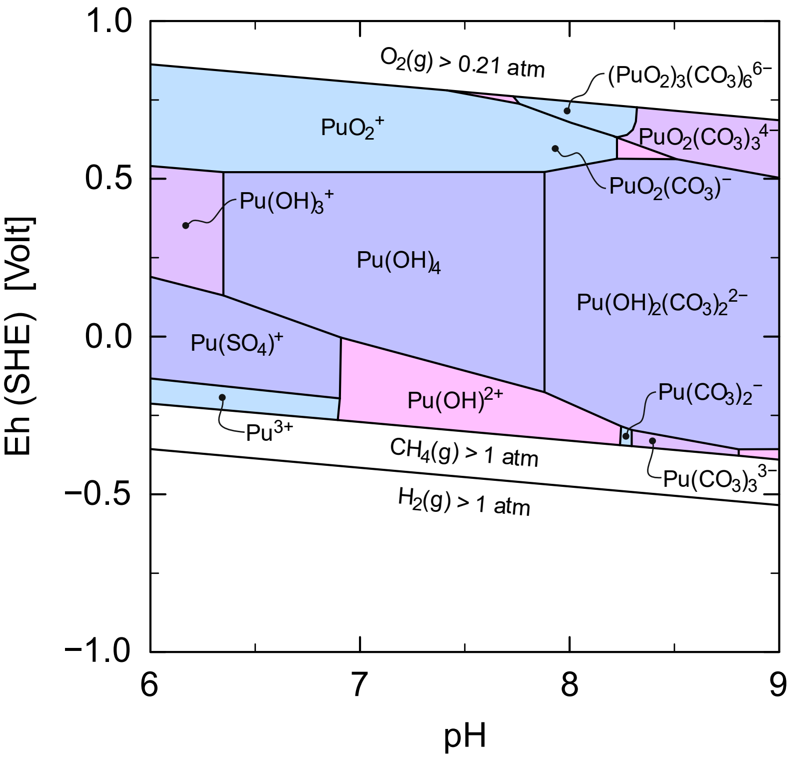

Figure S2: Predominance diagram for $2 \cdot 10^{-5}$ M Pu in Opalinus Clay pore water equilibrated with atmospheric CO_2. Graphic generated by PhreePlot (version 1.0) using PHREEQC[12] and the ThermoChimie database 12a-2023[13].

1.5 Experimental Setup and Sample Preparation for In-Diffusion Study

A summary of the employed experimental setup and sample preparation is given in Figs. S3 and S4.

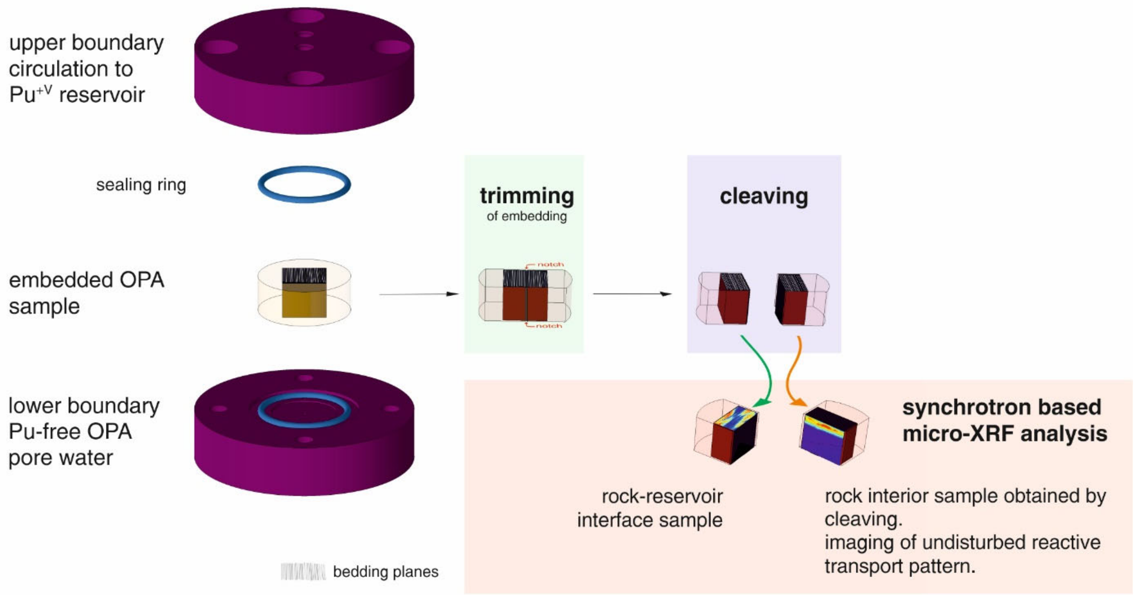

Figure S3: Sketch of experimental setup, sample preparation, and micro-XRF measurement geometries.

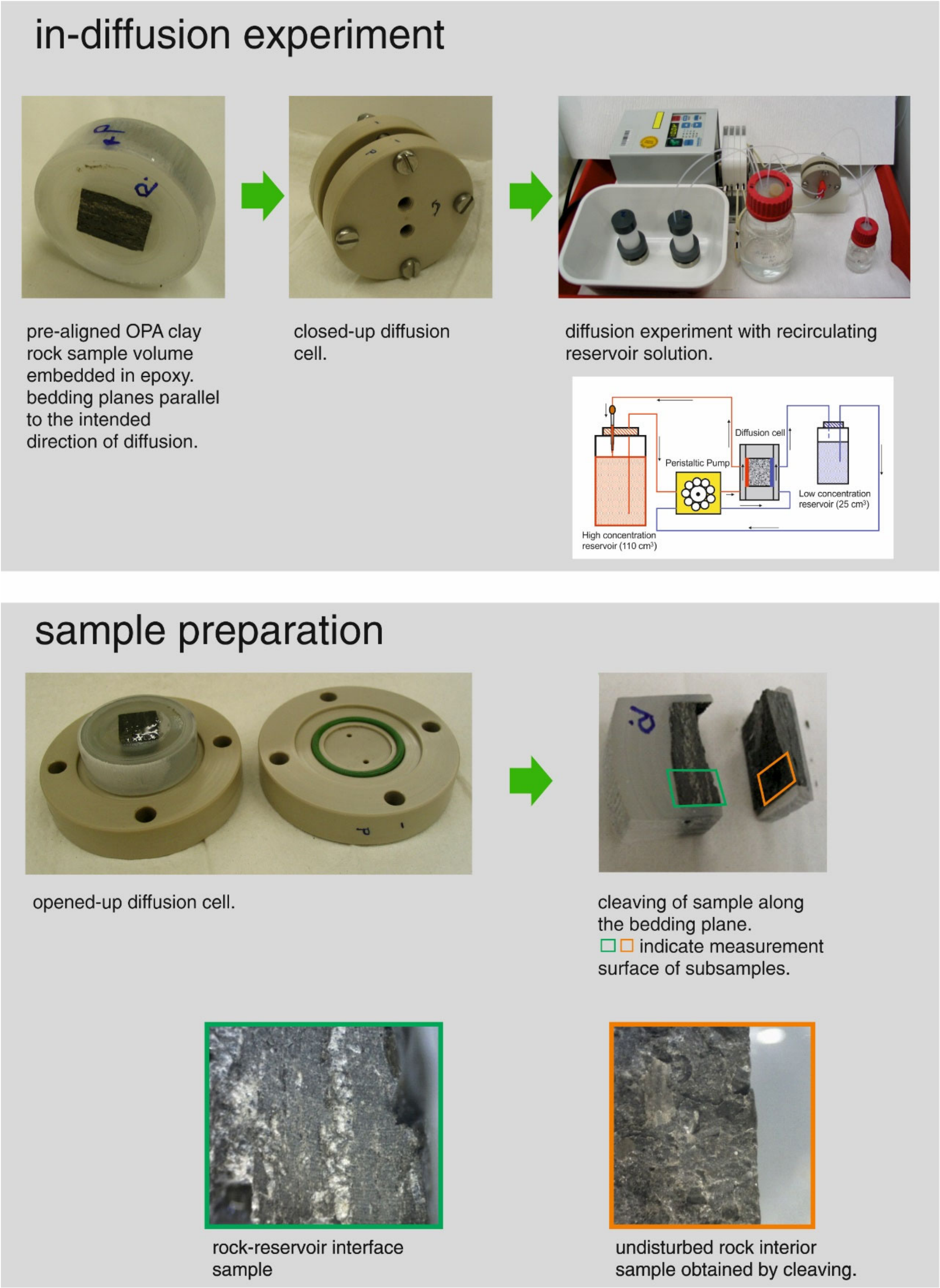

Figure S4: Experimental setup and sample preparation. Schematic representation of diffusion cell and solution circulation system (inset top panel) adapted from van Loon et al.[14]

1.6 Anaerob batch sorption study

Anaerobe OPA (BHE 24/2) was used for an anaerobe batch sorption experiment. The sorption of Pu^{+IV} on OPA powder was investigated using synthetic OPA pore water prepared under anaerobic conditions (Ar-atmosphere) at pH 7.6. The sorption experiments were carried out in 50 mL centrifuge tubes (Beckman Coulter, U.S.A.) with a total solution volume of 30 mL. The OPA powder (180 mg) was preconditioned for 72 h with oxygen-free OPA pore water. After the preconditioning, the OPA suspension was spiked with Pu^{+IV} from the ^{239}Pu stock solutions resulting in an initial Pu concentration of 10 µM. The $^{239}Pu(IV)$ stock solution was obtained from $^{239}Pu(VI)$ stock solution purified from its traces of ^{241}Am using an anion exchange chromatography. The tetravalent oxidation states of Pu was obtained by potentiostatic electrolysis and the purity was verified by UV/Vis spectroscopy at the characteristic absorption bands 470 nm[8]. After a contact time of 60 hours, the suspension was separated by a two-step centrifugation procedure, first at 4'025 g for 5 min (SIGMA 3K30, Sigma Laborzentrifugen GmbH, Germany), followed by 81'800 g for 1 h (Avanti J-30I, Beckman Coulter, U.S.A.). The Pu uptake was determined by measuring the Pu concentration in solution by liquid scintillation counting (LSC) using a home-built scintillation counter analyzing 1 mL sample in 10 mL scintillation cocktail Ultima Gold XR (PerkinElmer, U.S.A.). The Pu loading in the sample was 370 ppm. The powder sample was dried under anaerobic conditions for 3 days at 26 °C, grinded, loaded into a special polyethylene sample holder, sealed, and transported to the European Synchrotron Radiation Facility (ESRF) in a homemade special container filled with Ar gas.

For the anaerob batch sorption sample, a bulk EXAFS spectrum was collected at the Pu L_{III}-edge (18'057 eV) at 15 K in fluorescence mode using a 13-element Ge solid-state detector at the Rossendorf Beamline (ROBL BM20) of the European Synchrotron Radiation Facility (ESRF). For energy calibration the Zr K-edge (17'998 eV) spectrum of a Zr metal foil was measured simultaneously with each scan. The EXAFS analysis was performed with the software packages EXAFSPAK[15] and FEFF9.6[16]. The FEFF scattering phases and amplitudes for the Pu-O and Pu-Si interactions were calculated using a structural model based on density functional calculations of the incorporation of U(IV) in calcium-silicate-hydrate[17], where U was substituted by Pu. The modeling of the k^3-weighted EXAFS spectrum was performed in k-space (2.0 – 8.0 $Å^{-1}$) without a window function using seven independent parameters for the Pu-O and Pu-Si shells. Note that EXAFS cannot distinguish between Pu-Si and Pu-Al interactions.

2. Supplementary Experimental Results

2.1 Geochemical Heterogeneity and Reactive Transport Pattern

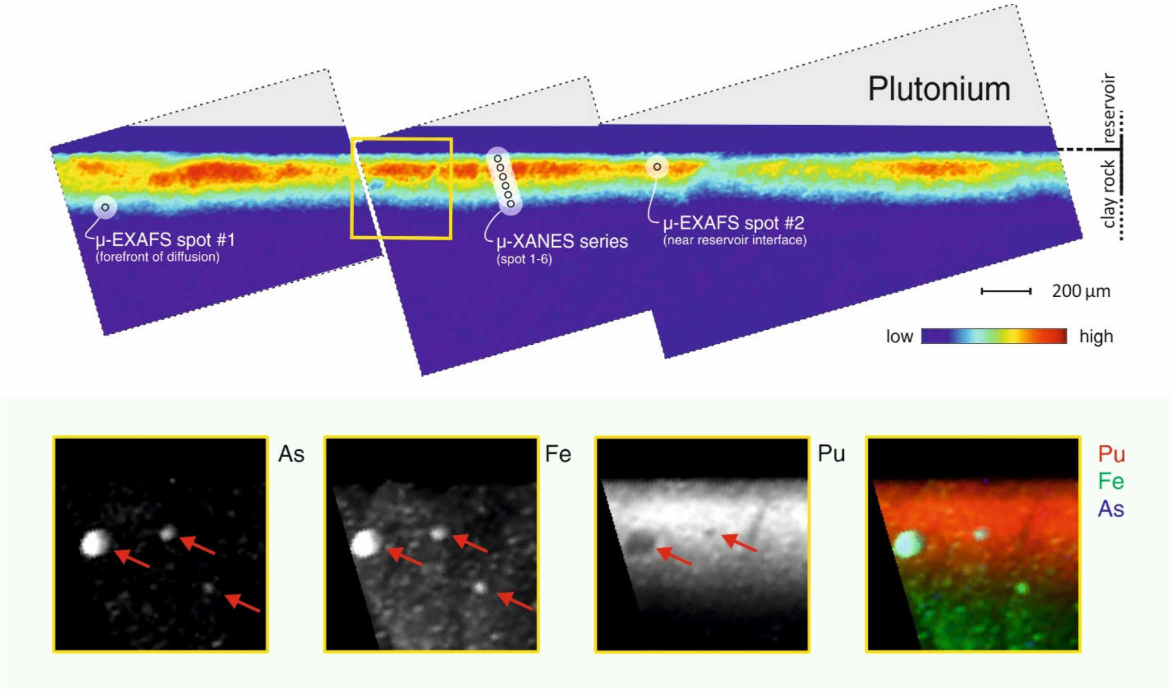

Figure S5: Elemental µ-XRF images detailing the impact of the geochemical heterogeneity on the reactive transport pattern. The yellow square in the top panel indicates the zoom area of the elemental images of Fe and Pu depicted below. Domains enriched in Fe and As (potentially arsenopyrites) reveal no reactivity towards Pu. Locations of µ-XANES series as well as µ-EXAFS measurements are indicated.

2.2 Spatially Resolved X-ray Absorption Spectroscopy (µ-XANES and µ-EXAFS)

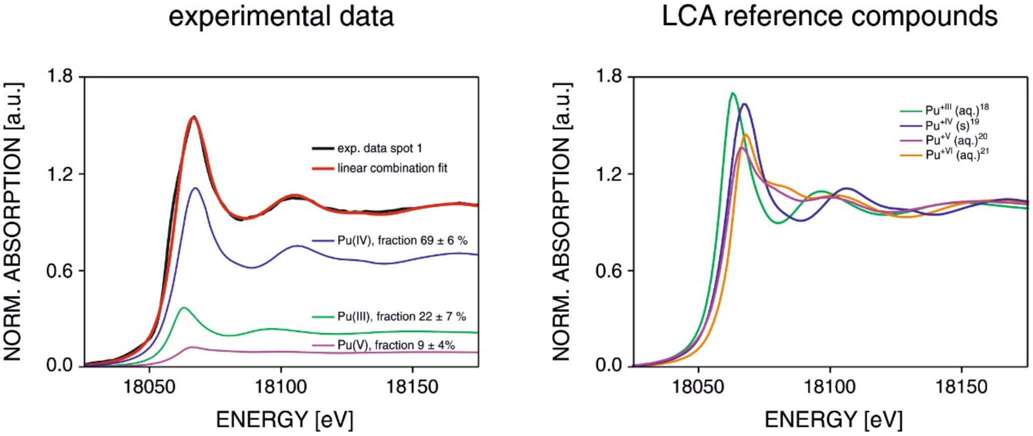

Figure S6: Normalized Pu L_{III}-edge µ-XANES spectra and related linear combination analysis. *left panel)* Spectrum recorded 50 µm from the reservoir – rock interface. *right panel)* reference spectra[18-21] used in the LCA.

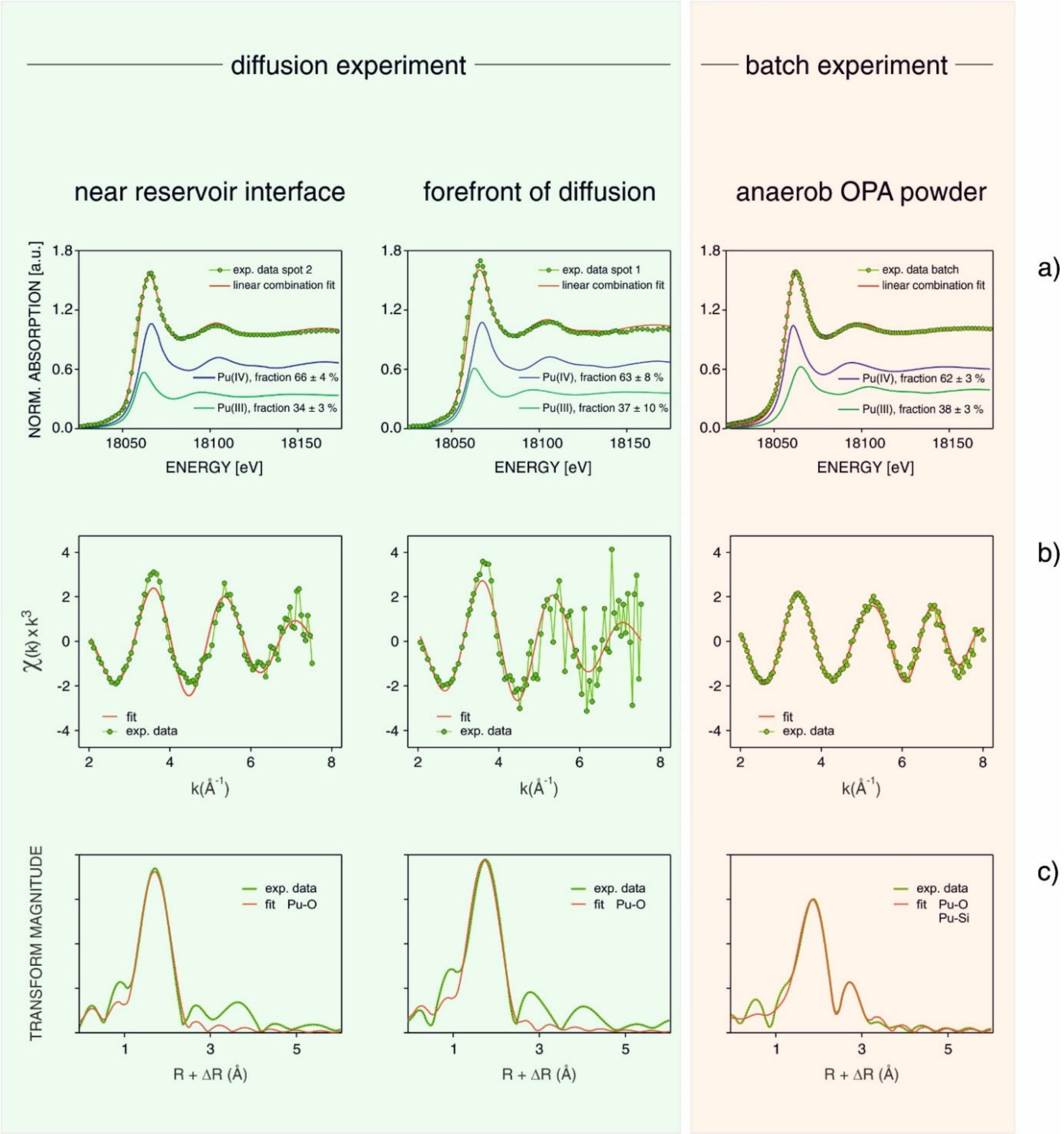

Figure S7: Chemical speciation of Pu after reacting with OPA determined by X-ray absorption spectroscopy. *Left panel*) Spatially resolved chemical speciation of Pu observed at specific locations within the reactive transport pattern developed by in-diffusion of Pu^{+V}. Measurement locations are indicated in Fig. S5. *Right panel*) bulk analysis of chemical speciation of Pu after reacting with anaerob OPA powder in an anaerob system. a) Pu L_{III}-edge XANES spectrum including the corresponding deconvolution by linear combination analysis. b) Pu L_{III}-edge k^3-weighted EXAFS spectrum and c) corresponding Fourier transform magnitude. Experimental data are compared to a single Pu-O shell fit (and a Pu-Si/Al contribution in case of the batch sample). Fitting results are summarized in Table S2.

Table S2a:

Summary Pu L_{III}-edge EXAFS first shell (Pu-O) fitting results

S_0^2 = 0.9, k_{min} = 2.0 $Å^{-1}$, k_{max} = 8.0 $Å^{-1}$, expected resolution 0.26 Å

**Diffusion Samples** (see Fig. S7)	Pu^{+III} [%]	Pu^{+IV} [%]	coordination number N	distance R [Å]	Debye-Waller σ^2 [$Å^2$]	ΔE [eV]	Ref.
diffusion forefront (spot #1)	37 ± 10	63 ± 8	8.9(2)	2.31(1)	0.022(4)	-0.3(9)	*this study*
near reservoir (spot #2)	34 ± 3	66 ± 4	7.2(6)	2.30(1)	0.018(2)	-0.9(6)	*this study*

Table S2b:

Pu coordination geometry, L_{III}-edge EXAFS, reference and literature samples

**Reference Samples**	Pu^{+III} [%]	Pu^{+IV} [%]	coordination number N	distance R [Å]	Debye-Waller σ^2 [$Å^2$]	ΔE [eV]	Ref.
Pu^{+IV} sorbed on anaerobe OPA (reference sample)	38 ± 3	62 ± 3	Pu-O: 7.9(3)<br>Pu-Si/Al: 1.9(3)	2.40(2)<br>3.17(1)	0.022(1)<br>0.006(2)	1.6(3)	*this study*
Pu^{+III} aq	100	[-]	9.2(2)	2.473(2)	0.0118(4)	0.5(2)	18
Pu^{+III} aq	100	[-]	8.6	2.50	0.0083	7.16	22
Pu^{+III} sorbed on magnetite, pH 8	100	[-]	8.1	2.49	0.0066	6.20	22
Pu^{+IV}aq	[-]	100	8-10	2.39			23
Pu^{+IV} sorbed on kaolinite, pH 4–9	.	.	Pu-O: 8<br>Pu-Si/Al: 2	2.28<br>3.62	0.0169<br>0.0091	0.28	24
PuO_2	[-]	100	8.0(5)	2.333(5)	0.0064(5)		19

2.3 Geochemical Domains and Pu Reactivity

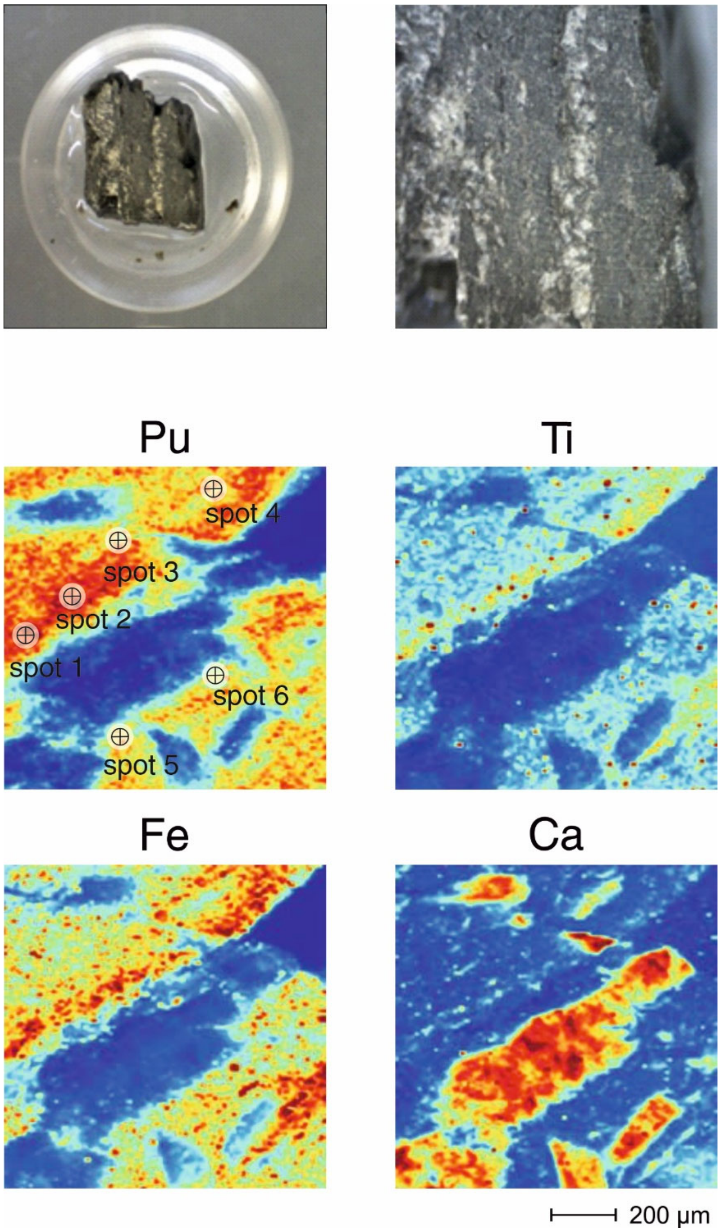

Figure S8: Elemental images obtained from the surface of the OPA piece in direct contact with the Pu spiked reservoir during the diffusion experiment. Shown are elemental distributions of Ca, Ti, Fe, and Pu. Chemical images were obtained by synchrotron-based µ-XRF. A distinctive geochemical (domain) heterogeneity is observed with a characteristic spatial length scale of few tens of microns. The field of view corresponds to 1’000 µm × 1’000 µm with a pixel size of 10 µm. The locations of the µ-XANES recordings shown in Fig. 5 are indicated in the Pu mapping.

3. Supplementary Information related to the Phenomenological Modelling

3.1 Processes ('phenomena') of reactive solute transport model

Table S3:

Processes of phenomenological model

	Reactions	Langmuir equilibrium constant, log K_{LM}	Rate forward	Rate backwards
Interface	$X + Pu^{+V} \Leftrightarrow X - Pu^{+V}$<br>$[X]_{tot}$ = 7.5 µmol/g	4.5	$1.2 \times 10^{+0}$ s^{-1} M^{-1}	3.8×10^{-5} s^{-1}
	$Y + Pu^{+IV} \Leftrightarrow Y - Pu^{+IV}$<br>$[Y]_{tot}$ = 30 µmol/g	6.5	$1.7 \times 10^{+3}$ s^{-1} M^{-1}	5.8×10^{-4} s^{-1}
	$Z + Pu^{+III} \Leftrightarrow Z - Pu^{+III}$<br>$[Z]_{tot}$ = 25 µmol/g	6.7	$1.2 \times 10^{+4}$ s^{-1} M^{-1}	2.1×10^{-3} s^{-1}
	Total site concentration ≅ 62.5 µmol/g			
Redox	$Pu^{+V} + e^{-}_{fast} \rightarrow Pu^{+IV}$<br>$Pu^{+IV} + e^{-}_{fast} \rightarrow Pu^{+III}$		3.5×10^{-1} s^{-1} M^{-1}<br>1.7×10^{-0} s^{-1} M^{-1}	
	$[e^{-}_{fast}]_{initial}$ = 15 µmol/g			
	$Pu^{+V} + e^{-}_{slow} \rightarrow Pu^{+IV}$<br>$Pu^{+IV} + e^{-}_{slow} \rightarrow Pu^{+III}$		1.2×10^{-5} s^{-1} M^{-1}<br>1.2×10^{-1} s^{-1} M^{-1}	
	$[e^{-}_{slow}]_{initial}$ = 50 µmol/g			

3.2 Model Parameters and Initial Guesses.

Table S4:

Parameters and initial guess values for phenomenological reactive (solute) transport modelling. Pu(V) diffusion parallel to bedding.

model parameter	modelling value	initial guess value or constraint	literature value	ref.
D_e [m^2/s]	$2.4 \cdot 10^{-11}$	$2.0 – 3.5 \cdot 10^{-11}$ #	$6.9 (\pm 1.1) \cdot 10^{-12}$ (Np^{V} perpendicular to bedding)	25
			$1.35 (\pm 0.2) \cdot 10^{-11}$ (HTO, perpendicular to bedding)	26
			$5.4 (\pm 0.1) \cdot 10^{-11}$ (HTO, parallel to bedding)	26
ε [-]	0.136	0.136	0.136<br>0.15 ± 0.01<br>0.15 ± 0.01	7<br>25<br>26
dry density [g/cm^3]	2.71	2.71	2.71<br>2.42	7<br>25
total site capacity [$mol\ kg^{-1}$]	0.0625	<0.1	0.082	27
Langmuir site X for Pu^{+V}	K_D: 0.23 [$m^3 \cdot kg^{-1}$] ◊<br>[X] = $7.5 \cdot 10^{-3}$ $mol \cdot kg^{-1}$	$[X] \cdot K_{LM} \cong 0.1$	≤ 1 [$m^3 \cdot kg^{-1}$]<br>Np(V) analogue K_D: 0.04 ± 0.01 [$m^3 \cdot kg^{-1}$]	28<br>29
Langmuir site Y for Pu^{+IV}	K_D: 95 [$m^3 \cdot kg^{-1}$] ◊<br>[Y] = $3.0 \cdot 10^{-2}$ $mol \cdot kg^{-1}$	85	K_D: 83 ± 33 [$m^3 \cdot kg^{-1}$]	29
Langmuir site Z for Pu^{+III}	K_D: 125 [$m^3 \cdot kg^{-1}$] ◊<br>[Z] = $2.5 \cdot 10^{-2}$ $mol \cdot kg^{-1}$	160	K_D: 160 ± 37 [$m^3 \cdot kg^{-1}$]	29

Estimate based on using Np^{+V} as analogue of Pu^{+V}. The K_d value for Np^{+V} reported by Wu et al. [25] measured perpendicular to bedding is corrected for anisotropy (parallel versus perpendicular to bedding).

◊ $K_D \cong [site] \cdot K_{LM}$ for $K_{LM} \cdot [Pu_{aq}] < 1$ (where K_{LM} corresponds to the Langmuir equilibrium constant)

References

1 Theurillat, T., Girardin, C., Badertscher, N. & Nussbaum, C. Drilling Campaigns of Phase 12 Drilling and Field Mapping of Drillcores Including Photo Documentation. pp. 302 (swisstopo, Wabern, Switzerland, 2004).

2 Bossart, P. & Thury, M. Research in the Mont Terri rock laboratory: Quo vadis? *Phys. Chem. Earth.* **32,** 19-31, doi:10.1016/j.pce.2006.04.031 (2007).

3 Thury, M. & Bossart, P. The Mont Terri rock laboratory, a new international research project in a Mesozoic shale formation, in Switzerland. *Eng. Geol.* **52,** 347-359, doi:10.1016/S0013-7952(99)00015-0 (1999).

4 Fröhlich, D. R. *et al.* Speciation of Np(V) uptake by Opalinus Clay using synchrotron microbeam techniques. *Anal. Bioanal. Chem.* **404,** 2151-2162, doi:10.1007/s00216-012-6290-2 (2012).

5 NAGRA. Projekt Opalinuston – Synthese der geowissenschaftlichen Untersuchungsergebnisse. Nagra Technical Report NTB-02-03 (NAGRA, Wettingen, Switzerland, 2002).

6 Pearson, F. J. *et al.* Mont Terri Project - Geochemistry of water in the Opalinus Clay formation at the Mont Terri rock laboratory. Report No. BWGG-5-E, (Federal Office for the Environment [FOEN], Bern, Switzerland, 2003).

7 Wersin, P., Mazurek, M. & Gimmi, T. Porewater chemistry of Opalinus Clay revisited: Findings from 25 years of data collection at the Mont Terri Rock Laboratory. *Appl. Geochem.* **138,** 12, doi:10.1016/j.apgeochem.2022.105234 (2022).

8 Cohen, D. The absorption spectra of plutonium ions in perchloric acid solutions. *J. Inorg. Nucl. Chem.* **18,** 211-218, doi:10.1016/0022-1902(61)80390-4 (1961).

9 Clark, D. L., Hecker, S. S., Jarvinen, G. D. & Neu, M. P. in *The Chemistry of the Actinide and Transactinide Elements* (eds L. R. Morss, N. Edelstein, J. Fuger, & J. J. Katz) Ch. 7, 813-1223 (Springer Netherlands, 2011).

10 Willberger, C., Amayri, S., Häussler, V., Scholze, R. & Reich, T. Investigation of the electrophoretic mobility of the actinides Th, U, Np, Pu, and Am in different oxidation states. *Anal. Chem.* **91,** 11537-11543, doi:10.1021/acs.analchem.9b00997 (2019).

11 Graser, C.-H. *et al.* Sensitive redox speciation of iron, neptunium, and plutonium by Capillary Electrophoresis hyphenated to Inductively Coupled Plasma Sector Field Mass Spectrometry. *Anal. Chem.* **87,** 9786-9794, doi:10.1021/acs.analchem.5b02051 (2015).

12 Parkhurst, D. L. & Appelo, C. A. J. PHREEQC (Version 3.3.5) - A computer program for speciation, batch-reaction, one-dimensional transport, and inverse geochemical calculations (2016, http://wwwbrr.cr.usgs.gov/projects/GWC_coupled/phreeqc/index.html).

13 Giffaut, E. *et al.* Andra thermodynamic database for performance assessment: ThermoChimie. *Appl. Geochem.* **49,** 225-236, doi:10.1016/j.apgeochem.2014.05.007 (2014).

14 Van Loon, L. R., Soler, J. M. & Bradbury, M. H. Diffusion of HTO, $^{36}Cl^-$ and $^{125}I^-$ in Opalinus Clay samples from Mont Terri - Effect of confining pressure. *J. Contam. Hydrol.* **61,** 73-83 (2003).

15 George, G. N. & Pickering, I. J. EXAFSPAK: A suite of computer programs for the analysis of X-ray absorption spectra (Stanford Synchrotron Radiation Laboratory., Stanford, 1995).

16 Rehr, J. J., Kas, J. J., Vila, F. D., Prange, M. P. & Jorissen, K. Parameter-free calculations of X-ray spectra with FEFF9. *Phys. Chem. Chem. Phys.* **12,** 5503-5513 (2010).

17 Chiorescu, I., Kremleva, A. & Krüger, S. On the sorption mode of U(IV) at calcium silicate hydrate: A comparison of adsorption, absorption in the interlayer, and incorporation by means of density functional calculations. *Minerals* **12,** 1541 (2022).

18 Schmeide, K., Reich, T., Sachs, S. & Bernhard, G. Plutonium(III) complexation by humic substances studied by X-ray absorption fine structure spectroscopy. *Inorg. Chim. Acta* **359,** 237-242, doi:10.1016/j.ica.2005.10.037 (2006).

19 Martin, P. *et al.* XAS study of $(U_{1-y}Pu_y)O_2$ solid solutions. *J. Alloys Compd.* **444,** 410-414, doi:10.1016/j.jallcom.2007.01.032 (2007).
20 Di Giandomenico, M. V. *et al.* Structure of early actinides(V) in acidic solutions. *Radiochim. Acta* **97,** 347-353, doi:10.1524/ract.2009.1620 (2009).
21 Reich, T. *et al. Plutonium. XAFS measurements of plutonium hydrates*, ESRF Highlights 1999, pp. 32-33, European Synchrotron Radiation Facility, (2000).
22 Kirsch, R. *et al.* Oxidation state and local structure of plutonium reacted with magnetite, mackinawite, and chukanovite. *Environ. Sci. Technol.* **45,** 7267-7274, doi:10.1021/es200645a (2011).
23 Conradson, S. D. *et al.* Higher order speciation effects on plutonium L_3 X-ray absorption near edge spectra. *Inorg. Chem.* **43,** 116-131, doi:10.1021/ic0346477 (2004).
24 Reich, T. *et al.* Application of XAFS spectroscopy to actinide environmental science. *AIP Conf. Proc.* **882,** 179-183, doi:10.1063/1.2644467 (2007).
25 Wu, T., Amayri, S., Drebert, J., Van Loon, L. R. & Reich, T. Neptunium(V) sorption and diffusion in Opalinus Clay. *Environ. Sci. Technol.* **43,** 6567-6571, doi:10.1021/es9008568 (2009).
26 Van Loon, L. R., Soler, J. M., Müller, W. & Bradbury, M. H. Anisotropic diffusion in layered argillaceous rocks: A case study with Opalinus Clay. *Environ. Sci. Technol.* **38,** 5721-5728, doi:10.1021/es049937g (2004).
27 Bradbury, M. H. & Baeyens, B. Predictive sorption modelling of Ni(II), Co(II), Eu(III), Th(IV) and U(VI) on MX-80 bentonite and Opalinus Clay: A "bottom-up" approach. *Appl. Clay Sci.* **52,** 27-33, doi:10.1016/j.clay.2011.01.022 (2011).
28 Amayri, S. *et al.* Interaction of Np, Pu, and Tc with a natural clay rock at higher salinities. (Institute of Nuclear Chemistry, University Mainz, Final Report of BMWi Project No. 02E10981, Mainz, Germany, 2016).
29 Amayri, S., Fröhlich, D. R., Kaplan, U., Trautmann, N. & Reich, T. Distribution coefficients for the sorption of Th, U, Np, Pu, and Am on Opalinus Clay. *Radiochim. Acta* **104,** 33-40, doi:10.1515/ract-2015-2409 (2016).
